# Expression profile and transcription factor binding site exploration of imprinted genes in human and mouse

**DOI:** 10.1186/1471-2164-10-144

**Published:** 2009-03-31

**Authors:** Christine Steinhoff, Martina Paulsen, Szymon Kielbasa, Jörn Walter, Martin Vingron

**Affiliations:** 1 Department of Computational Biology, Max Planck Institute for Molecular Genetics (MPIMG), Ihnestr 63-73, 14195 Berlin, Germany; 2 Biosciences, Genetics/Epigenetics, University Saarbrücken, 66041 Saarbrücken, Germany

## Abstract

**Background:**

In mammals, imprinted genes are regulated by an epigenetic mechanism that results in parental origin-specific expression. Though allele-specific regulation of imprinted genes has been studied for several individual genes in detail, little is known about their overall tissue-specific expression patterns and interspecies conservation of expression.

**Results:**

We performed a computational analysis of microarray expression data of imprinted genes in human and mouse placentae and in a variety of adult tissues. For mouse, early embryonic stages were also included. The analysis reveals that imprinted genes are expressed in a broad spectrum of tissues for both species. Overall, the relative tissue-specific expression levels of orthologous imprinted genes in human and mouse are not highly correlated. However, in both species distinctive expression profiles are found in tissues of the endocrine pathways such as adrenal gland, pituitary, pancreas as well as placenta. In mouse, the placental and embryonic expression patterns of imprinted genes are highly similar. Transcription factor binding site (TFBS) prediction reveals correlation of tissue-specific expression patterns and the presence of distinct TFBS signatures in the upstream region of human imprinted genes.

**Conclusion:**

Imprinted genes are broadly expressed pre- and postnatally and do not exhibit a distinct overall expression pattern when compared to non-imprinted genes. The relative expression of most orthologous gene pairs varies significantly between human and mouse suggesting rapid species-specific changes in gene regulation. Distinct expression profiles of imprinted genes are confined to certain human and mouse hormone producing tissues, and placentae. In contrast to the overall variability, distinct expression profiles and enriched TFBS signatures are found in human and mouse endocrine tissues and placentae. This points towards an important role played by imprinted gene regulation in these tissues.

## Background

Most genes in the mammalian genome are expressed from both parental alleles. Imprinted genes represent a minority of genes, which are transcribed from only one allele. While the molecular mechanisms underlying imprinting control have some commonalities, their individual expression control and expression patterns appear to vary in a developmental and tissue specific manner. A systematic investigation of their expression profiles may help to better understand the biological function and regulation of imprinted genes.

To date, approximately 100 genes with evidence for imprinting effects in either human or mouse are described [1]. Based on recent predictions, the number of mammalian imprinted genes may range between 100 and 600 genes [1-4], i.e. a substantial number of imprinted genes are already identified.

The imprinted expression of genes appears to be a rather conserved phenomenon in mammals [3]; i.e., genes that are found to be imprinted in one species are most likely imprinted in the other. This tenet, however, is not always fixed, as has been shown for the two orthologous man and mouse genes *L3MBTL *and *L3mbtl*. These genes each encode a polycomb protein. In human, the gene is frequently absent in patients with myeloid malignancies. Human *L3MBTL *has been shown to be paternally expressed due to monoallelic methylation [5,6] whereas mouse *L3mbtl *is not imprinted nor are its CpG islands differentially methylated [5].

Orthologous genes that are imprinted in human and mouse are most likely either maternally or paternally expressed in both organisms. Rarely, genes are oppositely imprinted such as the *ZIM2/Zim2 *genes: human *ZIM2 *is paternally expressed while mouse *Zim2 *is maternally expressed [7]. This phenomenon might be explained by the fact that the human *ZIM2 *gene shares 5' exons and a promoter with the likewise paternally expressed *PEG3 *gene, while mouse *Zim2 *appears not to do so [7].

Imprinted genes have been hypothesized to play a major role in the regulation of embryonic growth [8-10], to control placental function and to modulate the transport of nutrients from mother to embryo [11]. Indeed, a number of imprinted genes, such as *Ascl2, Phlda2, Peg10*, are indispensable for proper placental morphology and function while others are involved in nutrient supply regulation [11-14]. Additionally, there is strong evidence that imprinted genes control neurological development and function as well as energy homeostasis in postnatal stages of development and the adult [9,15,16].

Based on these various observations it seems likely that imprinted genes are tightly regulated in a developmental and tissue specific manner. While tissue specific expression profiles have been examined for some selected genes, no study on the entire class of imprinted genes has been performed so far. Furthermore little is known about the expression status in adult tissues compared to embryonic states.

In this study we performed a computational expression analysis of human and mouse imprinted genes in a variety of non-cancerous tissues using a set of existing systematic transcriptional profiling data [17]. In particular, we (1) compared the profiles of individual genes across tissues, (2) analysed the correlations of expression patterns in human and mouse, and (3) explored the role of predicted transcription factor binding sites in correlation to tissue specific expression. Our data provide new insights into the range and extend of expression, the tissue specific function and the regulation of imprinted genes in two mammalian species.

## Results

### Sets of imprinted genes selected for analysis

The Imprinted Gene Catalogue (IGC) [1,18] reports imprinted genes in various species, including human and mouse. We gathered information from the IGC on 62 genes for which solid experimental data on allele-specific expression was available [see Additional file 1] (see Methods for definition of exclusion criteria). Among these 62 genes, only 30 had been analysed in human as well as in mouse revealing that 26 of these are imprinted in both species. For one of these genes the status was only confirmed in human, for 3 genes only in mouse. Thus, of the genes analysed in both species 87% showed conservation of imprinting. For the additional 23 imprinted genes the imprinting status had only been analysed in mouse, and for additional 9 genes only in human.

### Tissue-specific expression patterns of imprinted genes

Using a publicly available gene expression dataset derived from microarray hybridisations we wanted to find out if imprinted genes form a subset of genes expressed in a particular fashion in human and mouse.

The raw microarray data were preprocessed and normalized as described in the Methods section. We confined the analysis to genes that were present on the respective expression arrays (GNF1M for mouse and HG-U133A for human) and which exhibited a confirmed imprinting status [see Additional file 1] in at least one species. For human, 29 imprinted genes met such criteria (of 35 genes with a confirmed imprinting status), and in mouse 43 (of 52 with a confirmed imprinting status). This list also includes genes reported to be imprinted in certain tissues only but not in others. As information on tissue specific imprinting is only available for some but not all imprinted genes the consideration of tissue-specific imprinting is not feasible for an unsupervised genome-wide approach. The array data did not allow distinguishing expression of the parental alleles. Our analysis could therefore only address the overall expression level. In the analysis of human gene expression we included 21 postnatal tissues and placental tissue. For the mouse, data sets for oocyte, fertilised egg and five embryonic stages were included. Unfortunately, such data was not available for the human.

**Figure 1:**
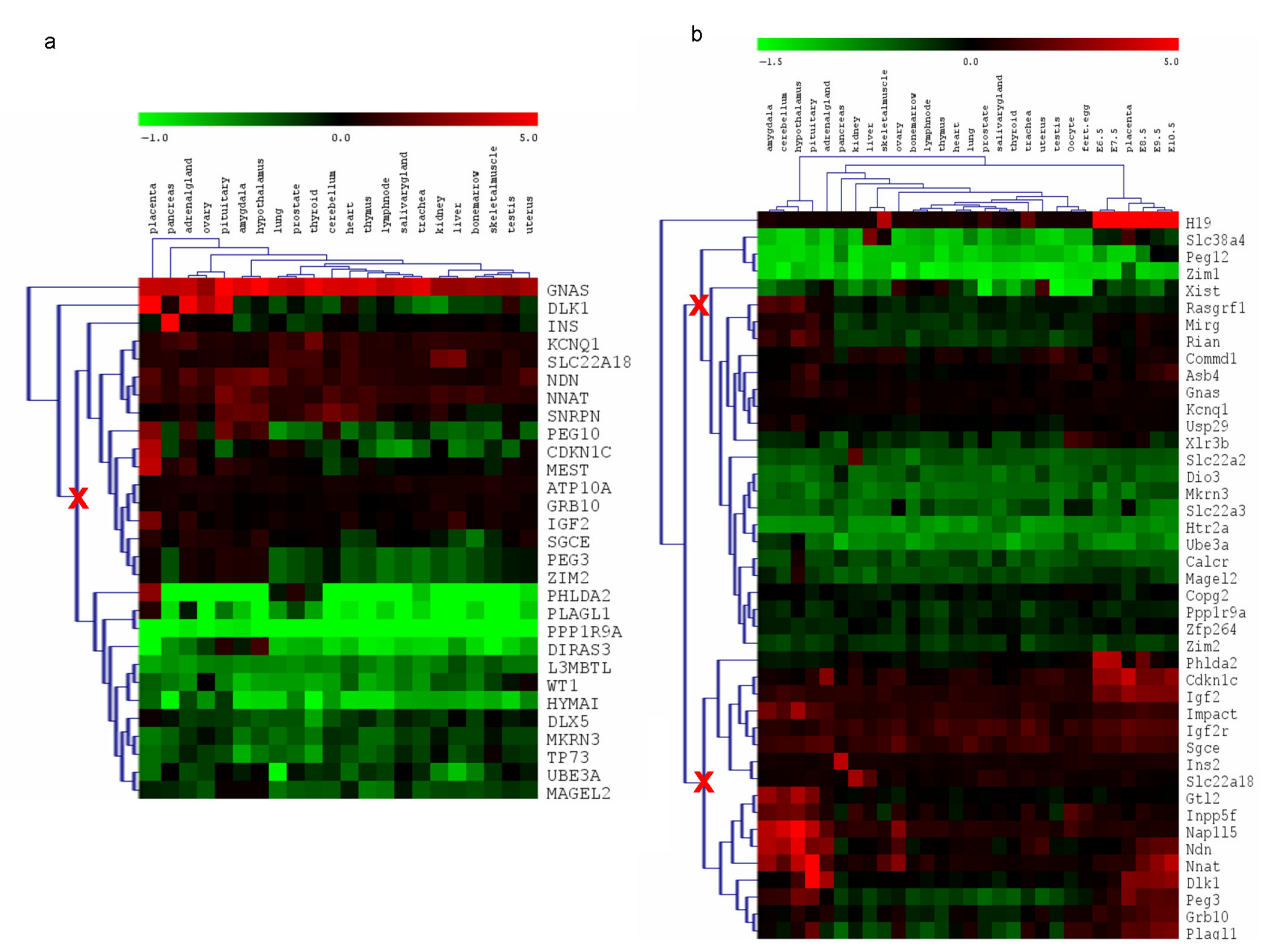
**Relative normalized expression levels of imprinted genes in human and mouse**. The figure summarizes the average (of normalized expression levels) of gene specific probes (annotated with the same gene name after normalization) of repeated array experiments. (a) A heatmap plot of biclustering (euclidean distance, average linkage) of human imprinted genes expression levels is shown. The third split within the genes tree is marked with a red cross. (b) A heatmap plot of biclustering (euclidean distance, average linkage) of mouse imprinted genes expression levels is shown. The third splits within the genes trees are marked by red crosses.

We first analysed the expression profiles of human and mouse separately. The aim was to identify tissues that differ considerably from other samples in terms of their expression profiles of imprinted genes. We performed biclustering (euclidean distance and average linkage). The resulting biclustered expression matrices from separate human and mouse expression analyses are shown in figure 1a and 1b.

For human, placenta forms a clearly separate branch (1^st ^split) in the clustered tree of tissues (Figure 1a). In the 2^nd ^split pancreas branches off (high influence of insulin), followed by a 3^rd ^split branch formed by pituitary, ovary, and adrenal gland. In the clustered tree of imprinted genes, *GNAS *clusters apart after the 1^st ^split followed by *DLK1 *in the 2^nd ^split. The expression of both genes differs significantly from the other genes in all tissues (p value < 10^-12 ^for all tissues). While *GNAS *was highly expressed in all tissues, *DLK1 *is significantly over-expressed in placenta, adrenal gland, ovary and pituitary and the high expression is relevant for the clustering of these tissues (see above). Regarding the 3^rd ^split, the remaining imprinted genes branch into two large clusters (Figure 1a). The first group, consisting of *INS, KCNQ1, SLC22A18, NDN, NNAT, SNRPN, PEG10, CDKN1C, MEST, ATP10A, GRB10, IGF2, SGCE, PEG3, ZIM2*, comprises genes expressed at largely median level. Among those *INS *stands out, that is remarkably over-expressed only in pancreas, the major insulin producing organ of the body. *IGF2, PEG10 *and *CDKN1C *are strongly (over)-expressed in placenta. The second cluster consists of *PHLDA2, PLAGL1, PPP1R9A, DIRAS3, L3MBTL, WT1, HYMAI, DLX5, MKRN3, TP73, UBE3A, MAGEL2 *with all genes showing a relative under-expression with respect to the tissue expression median. *PHLDA2 *and *PLAGL1 *are clearly downregulated in almost all tissues but strongly over-expressed in placenta. The genes that contributed most to the specific expression pattern of placenta and pancreas were those that were either strongly up or downregulated compared to their expression in other tissues. The placental expression pattern was dominated by *DLK1, PHLDA2, CDKN1C, MEST, PEG10 *and *IGF2*. For pancreas, *INS, DLK1, SNRPN, MEST KCNQ1 *and *HYMAI *were the most prominent genes. Finally, for the 3^rd ^split, a cluster which consisted of adrenal gland, ovary and pituitary, we applied random forest analysis to determine which genes contributed most to the formation of that cluster. These were *DLK1 *(mean standard error – MSE: 5.36%), *PPP1R9A *(4.61%), *HYMAI *(3.45%), *PEG3 *(2.3%), *MEST *(2.13%), *ATP10A *(2.09%), *ZIM2 *(1.99%). Applying a random forest analysis to the same tissues in mouse identifies *Dlk1 *(4.84%), *Sgce *(4.79%), *Kcnq1 *(2.11%), *Phlda2 *(1.91%), *Gtl2 *(1.76%), *Inpp5f *(1.41%), *Usp29 *(1.41%) as major contributors.

**Figure 2:**
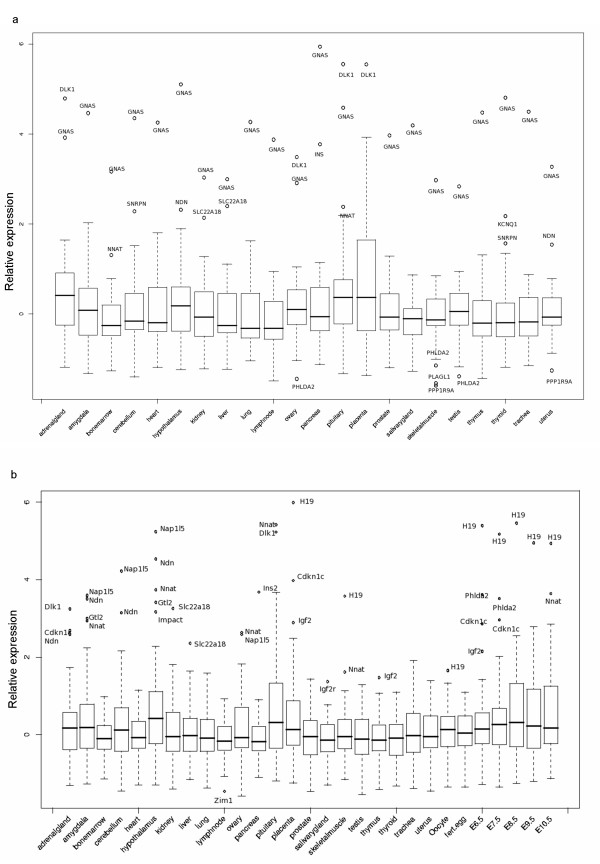
**Boxplots of relative expression levels**. As described in figure 1, normalized expression levels of imprinted genes in human (a) and mouse (b) are shown in boxplots. The x-axis displays the different tissues, and the y-axis indicates relative normalized expression levels. The horizontal bar in each box is the median of all relative expression levels of imprinted genes in human (a) and mouse (b), while the upper and lower boundaries of the box mark the first and third quantile. Whiskers show 1.5 times the box range, whereas circles denote values of data points that lie beyond the extremes of the whiskers (outlier). The respective gene names of outliers are displayed.

In mouse tissues the clustering has some similarities to human but also clearly distinct features. Pituitary and brain tissues branch off together at the 1^st ^split. This branching is predominantly caused by seven genes which we identified applying a random forest analysis as: *Gtl2 *(7.42%), *Rasgrf1 *(6.79%), *Nap1l5 *(6.57%), *Impact *(6.06%), *Inpp5f *(4.78%), *Mirg *(4.27%), *Rian *(4.08%). Applying a random forest analysis to the same brain tissues in human results in the following genes: *PEG3 *(7.44%), *PEG10 *(7.07%), *ZIM2 *(6.34%), *SNRPN *(5.49%), *NNAT *(5.12%), *SLC22A18 *(3.44%), *PPP1R9A *(2.81%). In the 2^nd ^split embryonic tissues separate from the adult ones (Figure 1b). The highest scoring genes for this cluster in a random forest analysis were: *Igf2 *(11.03%), *H19 *(10.50%), *Grb10 *(7.96%), *Cdkn1c *(7.34%), *Slc38a4 *(4.79%), *Plagl1 *(2.96%), *Peg3 *(2.63%). Most notably *Igf2*, *H19*, and *Cdkn1c *that dominate this branch lie in the BWS region and, together with *Phlda2*, are all highly expressed in embryonic tissues (Figure 2b). When omitting these four genes, from the clustering analysis the specific branching and clustering of embryonic stages is lost (data not shown). Among the postnatal tissues, adrenal gland splits off in 3^rd ^branch (as in 2^nd ^split in the human).

The clustering of imprinted genes in the mouse shows that a series of genes play a role for the branching into embryonic and brain specific clusters at the 1^st^/2^nd ^split. Aside from the predominant expression profile of *H19 *(1^st ^split, *H19 *is **not **represented on the human array), the remaining genes split into 2 clusters (2^nd ^split). One is the group characterised by moderate to high expression which splits into two clusters (3^rd ^split), where the cluster of *Gtl2*, *Inpp5f*, *Nap1l5*, *Ndn*, Nnat, *Dlk1*, *Peg3*, *Grb10 *and *Plagl1 *shows high expression in brain tissues. The second group is characterised by moderate to low expression and falls into two clusters according to the 2^nd ^split. Of these, the cluster of *Slc38a4*, *Peg12 *and *Zim1 *shows mainly low expression throughout the tissues.

Additional clustering (data not shown) by combination of Euclidean and Manhattan distances, respectively, was generated with either complete or average linkage. For mouse, the structures of the obtained trees were very similar to the ones shown in figure 1. The human clustering was found to be less stable (particularly applying Manhattan distance). However, placenta always separated in the first splits and in most analyses, pancreas separated in the 2^nd ^whereas adrenal gland and pituitary as well as amygdala and hypothalamus separated in the 2^nd ^or 3^rd^.

### Imprinted genes do not show prominent overexpression in distinct tissues

We next analysed whether imprinted genes on average are more strongly expressed in certain tissues compared to the non-imprinted genes present on the arrays (Figure 2a and 2b). For the analysis we sampled groups of non-imprinted genes (same number of genes as the examined imprinted gene group) 1000 times and compared their relative expression levels to the average expression of imprinted genes. These analyses were performed separately for each tissue. For human, the median expression levels of imprinted and non-imprinted genes were not significantly different (after multiple testing adjustment, i.e. Hochberg adjustment, p values ~ 0.64). In mouse, hypothalamus showed a slightly increased median expression compared to other tissues (p = 0.05).

We also compared the distribution of expression levels of imprinted and non-imprinted genes across tissues. Testing included either all non-imprinted genes on the array or randomly sampled sets. In both cases we observe similar distributions of standard deviations of expression levels across tissues between imprinted and non-imprinted genes (background) on the array [see Additional file 2]. Testing against randomly sampled gene sets the distributions of human and mouse standard deviations did not differ significantly from genomic background. Thus, the overall variability across tissues in relative expression of individual imprinted genes is not remarkably high with a few exceptions such as *DLK1 *and *INS *in human and *H19 *and *Ins2 *in mouse.

As a sum, imprinted genes show a median expression across tissues similar to non-imprinted genes. Except for a slight tendency in mouse hypothalamus, imprinted genes do not show a particular tissue-specific enrichment compared to the genome-wide average in either adult tissues or mouse embryonic tissues. In addition, imprinted genes did not show reduced or increased variability in tissue-specific expression levels. This suggests that on average imprinted genes tend neither to be expressed at almost constant levels in all tissues (like house keeping genes) nor to be only expressed in very few tissues.

We next tested whether any two tissues differ significantly. Adjusting for multiple testing in human tissues, no tissue pair reaches significance. In mouse, 60 tissue pairs out of 406 show a p value < 0.01. By chance we would expect 4 pairs with a p value of less than 0.01. Thus we observe approximately a 15 fold increase. Furthermore, pairwise comparison of embryonic tissues (fertilized egg, embryonic stages 6.5 – 10.5) with adult tissues resulted in 36 pairs with p value < 0.01 (out of 126). Hypothalamus is the tissue with the highest median expression level and shows significant over-expression in comparison to 14 tissues (out of 28 pairs). The detailed matrix is given in an additional table [see Additional file 3].

The biclustered expression matrices (Figure 1a and 1b) illustrate that several imprinted genes have conspicuous expression behaviour across tissues. *H19 *is such an outlier gene which in mouse is highly expressed at all embryonic stages and in skeletal muscle. Others, such as *GNAS/Gnas*, are strongly expressed in many tissues of one but not the other species pointing towards more general expression differences between human and mouse at this locus. Finally, in some tissues individual outliers show extensive differences in the relative expression between both species. An example is *Cdkn1c*, which is highly expressed in adrenalgland in the mouse but only moderately in human (Figure 1), although the general correlation between *CDKN1C *and *Cdkn1c *across all tissues is rather high (see below).

Overall, we observe that in pairwise comparisons imprinted genes are more highly expressed in mouse embryo than in adult tissues, especially bonemarrow, heart, lung, lymphnode, pancreas, prostate, salivarygland, testis, thymus, thyroid. The highest expression levels are observed for genes in the BWS region, namely *H19*, *Cdkn1c, Phlda2 *and *Igf2*. These genes dominate the biclustering of embryonic and placental samples in figure 1b whereas other genes behave rather inconspicuously at embryonic stages.

We also calculated the pairwise Pearson correlation coefficients for genes within particular imprinted regions, i.e. regions which at least contained three verified imprinted genes annotated to the same chromosomal band. The analysis shows that expression profiles show no more similarity among imprinted genes of a common cluster/chromosomal band than genes that reside in different regions (data not shown).

Already the biclustered expression matrices (Figures 1a and 1b) indicated that maternally and paternally imprinted genes, respectively, do not cluster together according to their tissue specific expression profiles. Calculation of Pearson correlation supports this notion showing that the parental origin of expression has no influence on tissue-specific expression profiles (data not shown). Still, overall, paternally expressed genes tend to be more highly expressed than maternal genes (for human p = 0.02, for mouse p = 0.04, t-test).

### Orthologous imprinted genes in man and mouse exhibit relaxed correlation of tissue specific expression

**Figure 3:**
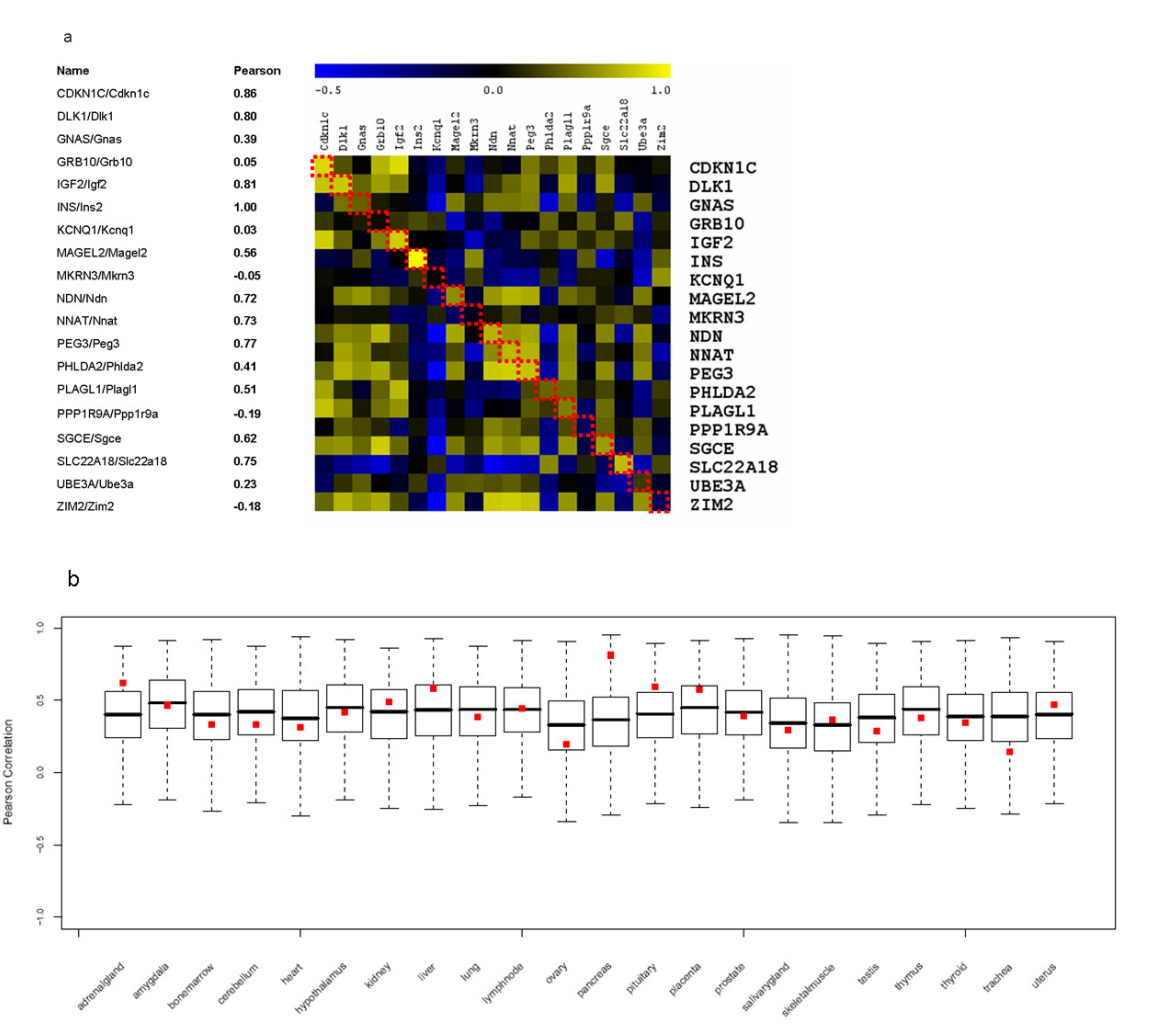
**Correlation of orthologous gene expression in human and mouse**. (a) Pearson correlation of normalized tissue-specific expression levels of orthologous imprinted genes in human and mouse. The figure shows Pearson correlation coefficients as 2 dimensional heatmaps of orthologous imprinted genes in human (vertical) and mouse (horizontal). The color coded scale is shown as a bar on top of the figure. Pearson correlation coefficients of orthologous gene pairs are marked on the dashed red diagonal line, and the respective values are given in the table aside. The correlations were calculated on normalized and averaged expression data of annotated genes (Figure 1 and Materials and Methods). (b) Pearson correlation of orthologous gene expression in human and mouse tissues. A set of 19 orthologous genes that were present on the human expression array as well as the mouse expression array was sampled 1000 times. Pearson correlation coefficients for each set in all tissues were calculated. The boxplot displays the resulting Pearson correlation coefficients as follows. The x-axis displays the different tissues, the y-axis lists the Pearson correlation coefficients. The horizontal bar in each box gives the median PCC of the sampled orthologous genes, while the upper and lower boundaries of the box mark the first and third quartiles. Whiskers show 1.5 times the box range. Red squares mark the PCC of imprinted orthologous genes.

We next investigated whether tissue-specific expression is correlated for orthologous imprinted genes in human and mouse (Figure 3a). Overall, orthologous gene pairs showed higher correlation than non-orthologous pairs (diagonal entries: median = 0.561; std (standard deviation) = 0.376; off diagonal entries: median = 0.093; std = 0.344). This difference is significant (Kolmogorov Smirnov test, p = 0.006; Wilcoxon test, p = 0.0006). Genes with the highest correlation values were: *INS/Ins2 *(pc (Pearson correlation) = 0.989); *CDKN1C*/*Cdkn1c *(pc = 0.861); *IGF2/Igf2 *(pc = 0.810); *PEG3*/*Peg3 *(pc = 0.774), and *DLK1*/*Dlk1 *(pc = 0.798). No correlation – i.e., a Pearson correlation of approximately zero – was observed for: *PP1R9A*/*Ppp1r9a*, *GRB10*/*Grb10*, *KCNQ1*/*Kcnq1*, and *MKRN3*/*Mkrn3*. In general, for imprinted orthologous gene pairs the correlation (pc = 0.56) across tissues is higher than genewise correlation of non imprinted orthologs (median pc = 0.20, 1000 times sampled sets of 19 genes). The values for gene pairs derived from the genomic background are in agreement with published results derived using slightly different methods [19].

In addition, we analysed if analogous tissues show similar expression profiles of orthologous imprinted genes in human and mouse. Out of a set of 8980 available orthologous genes we randomly sampled 1000 times genes of the same size as the imprinted gene set. For a given tissue we determined Pearson correlation coefficients comparing the relative expression profiles in human and mouse for each set of sampled genes. Thus, we derived 1000 Pearson correlation coefficients for the sampled gene sets and one for the imprinted gene set for each of the 22 tissues.

For 18 of 22 tissues, the correlation of expression of imprinted genes is in the 25% to 75% interquartile range (IQR) of randomly sampled orthologous genes (Figure 3b and [see Additional file 4]). In trachea, imprinted genes correlated slightly worse (median 0.388 for the random gene set and 0.142 for imprinted genes). In adrenal gland, pancreas, and pituitary, the correlation was stronger than in the set of random genes [see Additional file 4]. Although the correlation coefficient of placenta was between the 1^st ^and 3^rd ^quartiles (placenta differs from other tissues in its expression patterns of imprinted genes), it shows a clear tendency towards higher correlation in imprinted genes than in randomly sampled sets. In summary, the correlation values of human and mouse orthologous imprinted genes are not very different from those of randomly sampled non-imprinted orthologous genes (Figure 3b). In a few endocrine tissues we observe a strong expression correlation for orthologous imprinted genes. This finding is in line with previous individual expression reports on a few candidates in human and mouse [19,20].

### A few genes dominate expression profiles in distinct tissues

**Figure 4:**
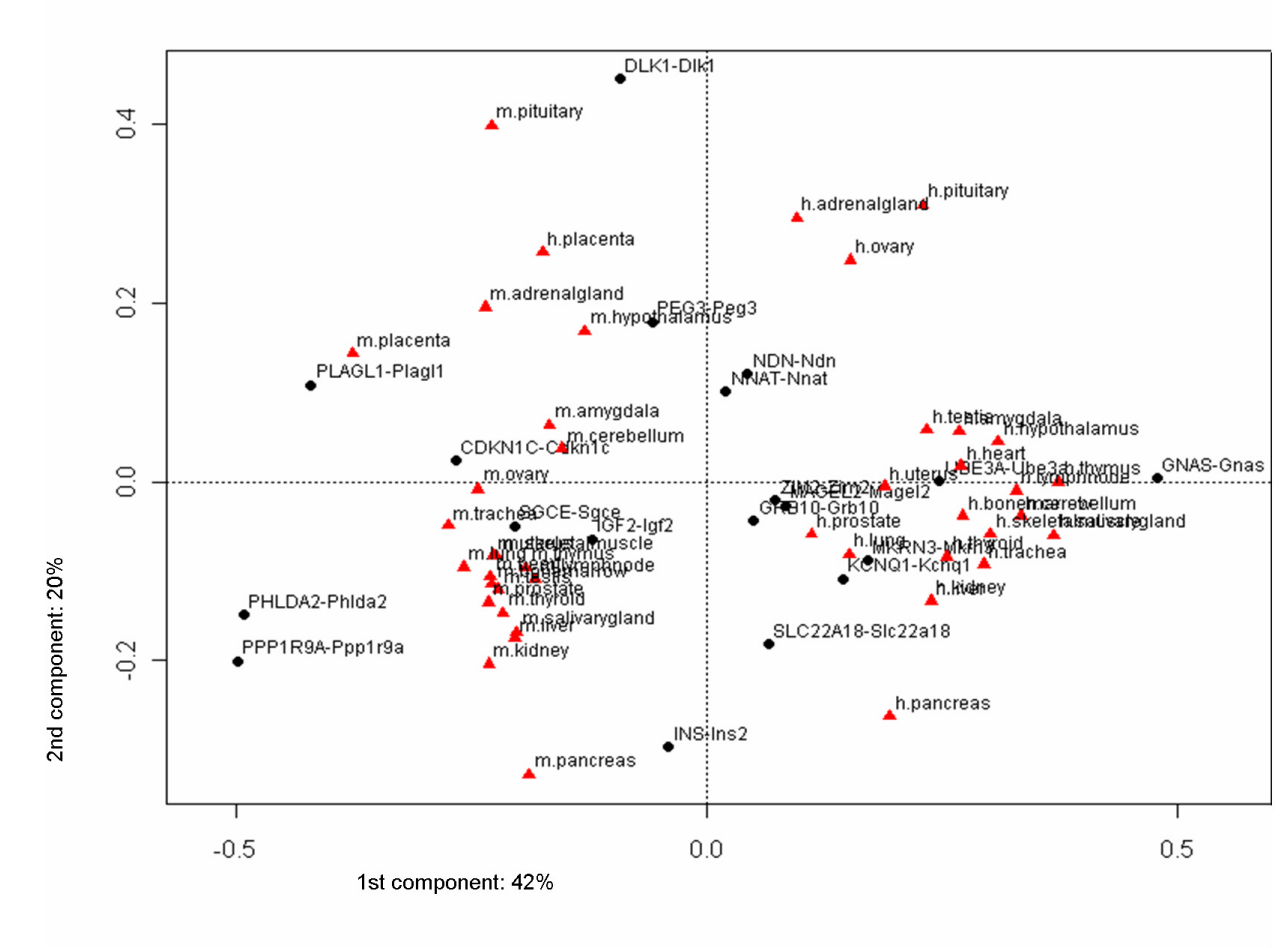
**Correspondence analysis of combined matrices of relative gene expression of orthologous imprinted genes**. Human and mouse imprinted gene expression for all tissues was analysed using correspondence analysis. In this figure, the first and second components are shown. Gene names are indicated, the respective vector end points marked with black spots. Localization of tissues is marked with red triangles and the names are given aside. Explained inertia of the first two components is: 41.89% and 19.59%, resp.

Using correspondence analysis, we next examined the relative contribution of individual genes to tissue specific expression profiles in human and mouse. Briefly, we applied a two way table (in our case relative expression values of 19 genes in 22 tissues each in human and mouse) correspondence analysis for describing/uncovering correspondence between rows (here genes) and columns (here tissues). Originally, the method was described by Berzerci [21]. For this, a high dimensional space of data points (genes, tissues) is constructed, such that the dimensions capture the variance explained by the given data in increasing order. Rotating the high dimensional data, they are projected onto a 2 dimensional planar in a way that maximal variance can be seen according to the first and second axis. In our application, this allows for studying associations between genes and tissues as shown in a two dimensional display (Figure 4). Objects (genes, tissues) with similar correlations are clustered together resulting in small angles, whereas dissimilar objects are separated from each other (large angle, e.g. different quadrants), furthermore, the larger the vector length the higher the information content. In figure 4, each point, i.e. tissue (red triangle) or gene (black dot), marks the direction and distance of a vector originating from the centroid. The appearance of vectors in the same quadrants and a closer angle distance between vectors reflects their relative association. While correspondence analysis allows us to visualize associations in complex matrices, it should be noted that there is no threshold to decide whether an association is strong or weak, the vectors describe relative associations, i.e. stronger or weaker than another.

Examples for such associations are mouse pituitary and human placenta which are associated with *DLK1/Dlk1 *and *PEG3/Peg3 *in the second quadrant. Further examples are (1) mouse placenta and *PLAGL1/Plagl1*, (2) mouse pituitary, human placenta and *PEG3/Peg3*, and (3) human adrenal gland, pituitary, ovary and *NNAT/Nnat*, *NDN/Ndn*.

Overall, however, the correspondence analysis (Figure 4) revealed highest variance between human and mouse tissues, with only human placenta being an exception (separated by the first component, which accounts for 41.89%). Thus, almost all human and mouse tissues were clearly separated (explained inertia of the first 5 components are: 41.89%, 19.59%, 10.74%, 9.55%, and 4.02%, respectively). A strong association was seen between *GNAS *and all human tissues except for pancreas, adrenal gland, ovary, pituitary, and placenta. *INS*/*Ins2 *showed a strong association with mouse pancreas but less so with human pancreas, while *SLC22A18 *showed a stronger association with human pancreas. *Dlk1 *was associated with mouse pituitary (Figure 4).

### Distinct sets of transcription factor binding sites correlate to tissue-specific expression patterns of imprinted genes

**Figure 5:**
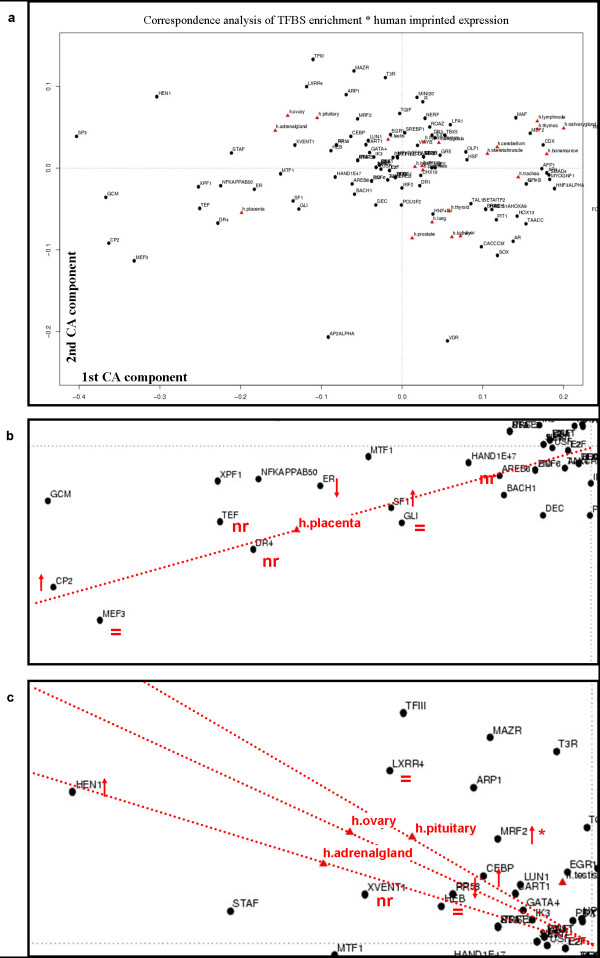
**Correspondence analysis of relative expression and transcription factor binding sites of human orthologous imprintedgenes**. (a) Human orthologous imprinted gene expression for all tissues as well as p-values for the 107 most prevalent TFBSs were analysed by multiplying the respective matrices. In this figure, the first (x axis) and second (y axis) components of correspondence analysis are shown. TFBS names are indicated by black spots, tissues in red triangles. (b) Zoom in the 3^rd ^quadrant of figure 5a. Placenta tissue is highlighted in red. A red dashed line marks the vector that defines the location of human placenta tissue in the correspondence plot. TFBS that show the smallest angle, i.e. highest association to human placenta were investigated for their relative expression in human placenta. A red arrow marks up-, downregulation compared to cellular background, equality signs stands for approximately expressed as cellular background and nr stands for not represented on the array. (c) Zoom for the 2^nd ^quadrant of figure 5 a. Pituitary, ovary and adrenal gland tissues are highlighted in red. A red dashed line marks the vector that defines the location of the three human tissues in the correspondence plot. TFBS that show the smallest angle, i.e. highest association to the three tissues were investigated for their relative expression in the same tissue. Caption is the same as in figure 5 b but for *MRF2 *the upregulation arrow and a star stands for the observation that *MRF2 *was found strongly upregulated in pituitary and ovary tissue but showed only slight upregulation in adrenal gland tissue.

Next, we questioned whether tissue-specific expression of imprinted genes is regulated by a well defined set of transcription factors. Addressing a possible connection between tissue-specific imprinted gene expression and predicted transcription factor binding sites (TFBS), we again applied a correspondence analysis. We computed a matrix of predicted TFBSs (based on a TRANSFAC pattern) in the upstream region of the respective genes (scored by p values). By multiplying this score matrix with the matrix of relative expression strength of the respective genes we derived a score matrix that relates human tissues and TFBS enrichment based on the studied set of imprinted genes. For visualization we performed a correspondence analysis (Figure 5).

The analysis reveals that imprinted genes expressed in human placenta are more frequently associated with binding sites for XPF1, NFKappaB50, ER, MTF1, SF1, GLI, TEF, DR4, MEF3 and CP2. In fact, *XPF1, TEF, MTF1, SF1*, *GLI, MEF3 and CP2 *were also present on the expression array and we found *XPF1, MTF1, SF1*, *GLI *and CP2 to be upregulated (defined as higher-than-median expression plus 1 SD) in human placenta (Figure 5b). We further extracted human placenta-expressed genes from the expression dataset and explored whether the same group of transcription factors binding sites were enriched. In fact, all TFBSs except for XPF1 and MEF3 showed significant enrichment. NFKappaB50 had a p-value of 4.3*10^-44^, TEF 7.3*10^-69^, DR4 4.1*10^-7^, ER 0.002, MTF1 9.9*10^-57^, SF1 1.2*10^-11^, GLI 5.9*10^-20 ^and CP2 4.4* 10^-41 ^while XPF1 and MEF3 had a p-value of 0.4. After adjustment for elevated GC content of the upstream regions of imprinted genes, binding sites for NFKappaB50, TEF, MTF1, SF1 and CP2 still showed clear enrichment, while binding sites for DR4, ER, and GLI showed no enrichment. In summary, NFKappaB50, TEF, MTF1, SF1 and CP2 displayed placenta-specific TFBS enrichment. Binding sites for XPF1 were significantly enriched in the upstream regions of imprinted genes but not in those of placenta-expressed genes, with or without GC content adjustment.

In mouse, the same TFBSs were significantly enriched as in the human set, namely for NFKappaB50, TEF, MTF1, SF1 and CP2 (even after adjustment for elevated GC content). In addition, DR4 and GLI binding sites also showed significant enrichment. Overall, the results were comparable between human and mouse imprinted genes.

Prominent examples of tissues specific TFBS associations in imprinted genes can also be observed for adrenal gland, pituitary and ovary (2^nd ^quadrant in Figure 5a, and Figure 5c). As for the clustering of human imprinted genes expression, (Figure 1a) the multiplied dataset consisting of TFBS enrichment and expression displays a very pronounced cluster of pituitary, adrenal gland and ovary. The strongest associations to these tissues as can be directly read from figure 5c are HEN1, LXRR4, MRF2, CEBP, RP58, HEB and XVENT1 binding sites. While *XVENT1 *is not represented on the array, *HEN1*, *MRF2 *and *CEBP *show a very pronounced upregulation in ovary and pituitary (at least two fold upregulation compared to the cellular background). For adrenal gland these are *HEN1 *and *CEBP *while *MRF2 *is at least 1.4 fold upregulated.

## Discussion

A general observation of our analysis is that imprinted genes are expressed in a broad range of adult tissues and placenta in human and mouse. For most tissues, the correlation of expression patterns of imprinted genes in human and mouse is not pronounced and does not differ from that of other randomly selected orthologous genes. Furthermore, the organization of imprinted genes into genomic clusters does not coincide with coordinated tissue-specific gene expression patterns within these genomic regions. Besides the overall "inconspicuous" expression behaviour of imprinted genes we observe particular expression patterns of subsets of imprinted genes in certain human and mouse tissues. Tissues with distinct expression profiles such as placenta, adrenal gland, ovary and pituitary show a remarkable correlative association with distinct TFBSs in the promoter regions of imprinted genes. As very little is known about transcription factors that regulate imprinted genes the identified associated factors are good candidates for experimental studies on tissue-specific regulation of imprinted genes. In addition, various imprinted zinc finger protein genes have been identified that may act as transcription factors. Among these is *PLAGL1*/*Plagl1 *that is strongly associated with murine placenta and apparently possesses the potential to regulate *Igf2 *and *H19 *[22], and also *PEG3/Peg3 *that is associated with mouse pituitary and human placenta.

In line with the parental conflict hypothesis [23], embryonic development and placental phenotypes are associated with imprinting mutations and imprinted gene expression [24-26]. We observe that imprinted genes do not show a generally stronger correlation of tissue-specific expression patterns in human and mouse and hence are unlikely to form a "uniform class" of genes whose functions are restricted to the same tissues and (embryonic) stages in both species. It will be interesting to extend detailed tissue specific comparisons to mouse/human embryonic stages to find out if distinctive tissue specific patterning is observed during prenatal development. So far our analysis (confined to total embryo and placenta expression) indeed suggests that the overall expression of imprinted genes in embryo and placenta is distinct from adult tissues. However, this distinction results from an exceptional expression of a rather small group of imprinted genes, with most being located in the BWS region.

One major observation of our analysis is that species specific subsets of imprinted genes form groups with pronounced expression correlation in adrenal gland, pancreas, and pituitary in human and mouse. In the correspondence analysis plots, these three organs and also placenta separate from the vast majority of tissues. All four organs (adrenal gland, pancreas, pituitary, placenta) play prominent roles in the energy metabolism of mammals: the placenta acts as the key organ in nutrient transfer between mother and embryo, and the adrenal gland, pancreas, and pituitary secrete factors, such as insulin and hormones, that play major roles in carbohydrate metabolism [16]. Thus, coordinated imprinted gene expression in these organs may be of importance for balanced physiological pathways in energy supply. This supports the parental conflict hypothesis to some extent, which proposes imprinted genes as regulators of growth and maternal nutrient supply. Interestingly, we observe a trend towards higher expression levels of paternally expressed genes. It will be interesting to find out if these genes are particularly involved in regulating nutrient demand or other discussed functions of these endocrine tissues, such as stress response, salt and fluid balance.

Several imprinting syndromes suggest that (some) imprinted genes fulfil important neuronal functions and hence may be particularly expressed in certain neuronal tissues [27]. Although hypothalamus, amygdala and cerebellum cluster apart from other tissues in mouse (biclustering analysis), they do not separate from other tissues in human. Also, the correspondence analysis separates brain tissues only marginally from other tissues in mouse. Hence, at least for human and mouse, particular brain-tissue specific expression profiles are likely to be species specific. In the human, the lack of particular profiles in certain brain tissues (such as hypothalamus, cerebellum, amygdala) may even indicate that here imprinted control might be confined to only a few genes such as *NDN *and *SNRPN*. However such extreme interpretations have to be taken with great caution given the fact that some genes such as *GNAS *or *DLK1 *show a strong and broadly extended expression pattern in most human tissues including brain.

## Conclusion

In summary, imprinted genes are expressed in a broad range of tissues in the adult of human and mouse. According to their overall expression pattern they do not form a particular class of autosomally expressed genes. However, subsets of imprinted genes are strongly expressed in the pituitary, adrenal gland, pancreas and placenta. Hence particular expression patterns are found in tissues regulating hormonal and nutritional homoeostasis in both human and mouse. Such correlated expression and the enrichment of tissue specific TFBSs suggest mechanisms of co-regulation of selected imprinted genes in organs/tissues controlling hormonal pathways and growth physiology in both species.

## Methods

### Gene Selection

Imprinted genes of human and mouse were downloaded from the Imprinted Genes Catalogue (IGC, 11/2007) [18]. For some of these genes, there were conflicting reports about their imprinting status. A number of these genes were biased towards the maternal allele only in placenta. Because this organ is composed of maternal and embryonic tissue, it is difficult to distinguish whether maternal expression of genes is caused by expression in the maternal sections or by imprinted expression. Therefore, we neglected from the analyses genes for which conflicting data have been reported, as well as genes for which the only evidence for possible imprinting effects is an expression bias toward the maternal allele in placenta. Furthermore, antisense transcripts were excluded from analysis since their genomic organisation is often insufficiently defined and are often not easy to be distinguished from sense transcripts, especially in hybridisation experiments. Small RNAs such as microRNAs and snoRNAs were also removed from the analyses since they are often part of longer transcripts. The resulting, manually curated list of 62 imprinted genes is given in an additional file [see Additional file 1]. Corresponding Ensembl Identifier, potential HG-U133A Identifier, GNF1M Identifier, and Gene Symbols were determined based on the Gene Names given in the IGC list using Ensembl BioMart . 11 genes were found only in one species; i.e., we failed to identify an ortholog of sufficient identity in the other species. In total, the list encompasses 36 human and 52 murine imprinted genes.

When examining imprinted gene expression in each species separately (human and mouse), we only used genes that had a confirmed imprinting status (i.e., those that are marked with a tick in an additional table [see Additional file 1]) that were present on the respective expression array (that is, GNF1M for mouse and HG-U133A for human). For human, there were 29 imprinted genes, and 43 for mouse. For human-mouse comparison, we used those orthologous genes (see below) that (a) were present on both arrays and (b) showed verified imprinting status in human and mouse. In total, we analysed 19 genes. For our correlation analysis, the Pearson correlation coefficients were calculated.

### Expression analysis

For gene expression analysis, we used the expression data reported by Su and colleagues [17]. This data has been studied with various foci several times and proven its validity for addressing expression related studies. Nevertheless, to our knowledge it has not been studied in the context of imprinting so far. The human sample dataset was based on the commercially available HG-U133A array; for mouse samples, a custom-designed array was used. Raw data were downloaded and subjected to pre-processing as follows: raw probe-set intensities were normalized using the calibration and variance stabilization method (vsn) [28]. Using this procedure, the variance of normalized probe intensities was approximately independent of their expected absolute expression levels.

For each experiment, it was assumed that the majority of gene expression was not differential with regard to all other experiments on the same array type. Parameters for the vsn model were estimated for a random subset of 50% of the probes and then used to transform the entire array. Probe-set intensities of each probe set were summarized by applying the median polish method [29] after normalization. Herein, for each probe-set, a robust additive model was fitted across the arrays. For further analysis, only those experiments that were annotated with the following tissue names for both human and mouse expression experiments were considered: adrenal gland, amygdala, bone marrow, cerebellum, heart, hypothalamus, kidney, liver, lung, lymph node, ovary, pancreas, pituitary, placenta, prostate, salivary gland, skeletal muscle, testis, thymus, thyroid, trachea, and uterus. For mouse we additionally studied embryonic stages 6.5, 7.5, 8.5, 9.5, 10.5, fertilized egg and oocyte. These stages were not available for human.

Because absolute expression levels were not appropriate for comparison between species, even after normalization, we used relative expression values with respect to the genome-wide profile of the same cell type in each species; i.e., we subtracted the cell type-specific background expression from each normalized (transformed scale) gene expression value. Repeated measurements were averaged. We called the resulting expression values 'normalized expression levels' or to be more precise 'normalized relative expression levels'. We also checked whether relative expression and absolute expression differ strongly which is not the case (data not shown).

### Visualization of expression profiles and statistical analyses

Biclustering and generation of heatmaps as shown in figure 1 and visualization of Pearson correlation of human/mouse relative expression as shown in figure 3a were done using TM4 [30]. For biclustering, Euclidean distance and average linkage were chosen. We confirmed that top splits remained approximately the same when changing the distance and clustering method. Therefore we restricted the analysis shown here to Euclidean distance and average linkage. All further calculations and statistical analyses were performed using the statistical language R [31] and packages from Bioconductor [32]. Correspondence analysis was calculated using the package "ca" [33]. For classification of genes that define subclusters of tissues (i.e. brain and embryonic tissues) we applied random forest tests [34] and used the package "randomForest". Further statistical tests were conducted using the Base package.

### Definition of orthologous genes

Using Ensembl BioMart, all orthologous genes in human and mouse that were (a) annotated with orthology type "one2one" and (b) present on the HG-U133A Affymetrix chip and on the GNF1M chip were determined. Probe-sets that were annotated to these genes were identified, in total 8980 genes. Of these, the gene sets that had the same size as the orthologous imprinted genes were randomly sampled 1000 times.

### Definition of upstream regions and sequence retrieval, and examination and identification of transcription factor binding sites

The transcription factor binding sites (TFBSs) were predicted using the method developed by Rahmann and colleagues [35]. We used Transfac 9.4 database to obtain position specific scoring matrices (PSSMs) preferentially recognized by transcription factors. In order to reduce the amount of false or overlapping predictions we narrowed the whole PSSM set to 125 non-redundant, high-quality, vertebrate matrices.

As putative promoters we defined the sequences located from 2000 bp upstream to 2000 bp downstream around ENSEMBL predicted transcription start sites (TSSs) of genes (according to ENSEMBL database, version 42). We scanned each of the putative promoters for nucleotide patterns matching the PSSMs. A match was accepted when the similarity score was above a threshold score defined as obtaining one single false positive prediction per 500 nt with a probability of 0.01 (see [35] for details). The above criterion yielded 175 TFBSs for 19 studied human imprinted genes. The p-values corresponding to these predicted TFBSs were then used as input for the correspondence analysis as described above.

## Authors' contributions

CS, MP, JW and MV designed the overall project. CS retrieved data used in this paper, performed the expression analysis, correspondence analysis and further statistical analysis. SK performed transcription factor binding site exploration. CS, MP, JW and MV wrote the manuscript. All authors read and approved the final manuscript.

## Supplementary Material

Additional file 1 **Genomic localization, imprinting status, availability of expression data of 62 imprinted genes from Imprinted Genes Catalogue (IGC)**. ^a ^gene names of imprinted genes in human and mouse (in brackets). ^b ^genomic localization of imprinted genes in human and mouse (in brackets). ^c ^imprinting status: tick = imprinting status confirmed, ? = questionable imprinting status, N = not imprinted, NO = no orthologous, AS = antisense. ^d ^Expressed allele: M = maternal, P = paternal, P/M = opposite imprinting status in human and mouse, i.e. *COPG2/Copg2 *in human has been reported to be paternally expressed but this finding is disputed, while it is maternally expressed in mouse. *ZIM2/Zim2 *is paternally expressed in human while maternally in mouse. For *GRB10/Grb10 *the imprinting status is dependent on the isoform. ^e ^Imprinting status in human and mouse: tick = respective gene imprinted in human and mouse. ^f,g ^Gene is represented on the expression array studied in this paper Click here for file

Additional file 2 **Distribution of standard deviation across tissues**. The left figure displays the distribution for human data whereas the right one shows mouse data. The background distribution, consisting of all genes present on the array but imprinted genes of standard deviation for each gene across tissues is marked in black. The standard deviation of imprinted genes is displayed as red histograms. Click here for file

Additional file 3 **Pairwise comparison of mouse tissues**. Here we report the p values resulting from two sample t-test of each pair of mouse tissues. For this based on the relative expression levels of imprinted genes studied in mouse we calculated for each two tissues the p value of student's t test. Click here for file

Additional file 4 **Comparison of tissue-specific expression of randomly sampled and imprinted orthologous genes**. The median, mean, 1^st ^and 3^rd ^quartile of 1000 times randomly sampled non imprinted orthologous genes for each tissue are shown in columns 2–5. In column 6 the Pearson correlation coefficient comparing randomly sampled genes with imprinted genes relative expression is displayed, column 7 gives the respective quartile according to the randomly sampled distribution and column 8 indicates with a star the cases, where the Pearson correlation exceeds the 1^st ^or 3^rd ^quartile. a median, mean, 1st quartile and 2nd quartile Pearson correlation expression of 1000 times random sampling of non imprinted orthologous genes in each tissue. b Pearson correlation of imprinted genes expression comparing human and mouse in each tissue. Click here for file
