# Supplementary figures and images for: Expression profile and transcription factor binding site exploration of imprinted genes in human and mouse
Source: BMC Genomics. 2009 Mar 31;10:144. doi: 10.1186/1471-2164-10-144 (PMC2671526; doi:10.1186/1471-2164-10-144)

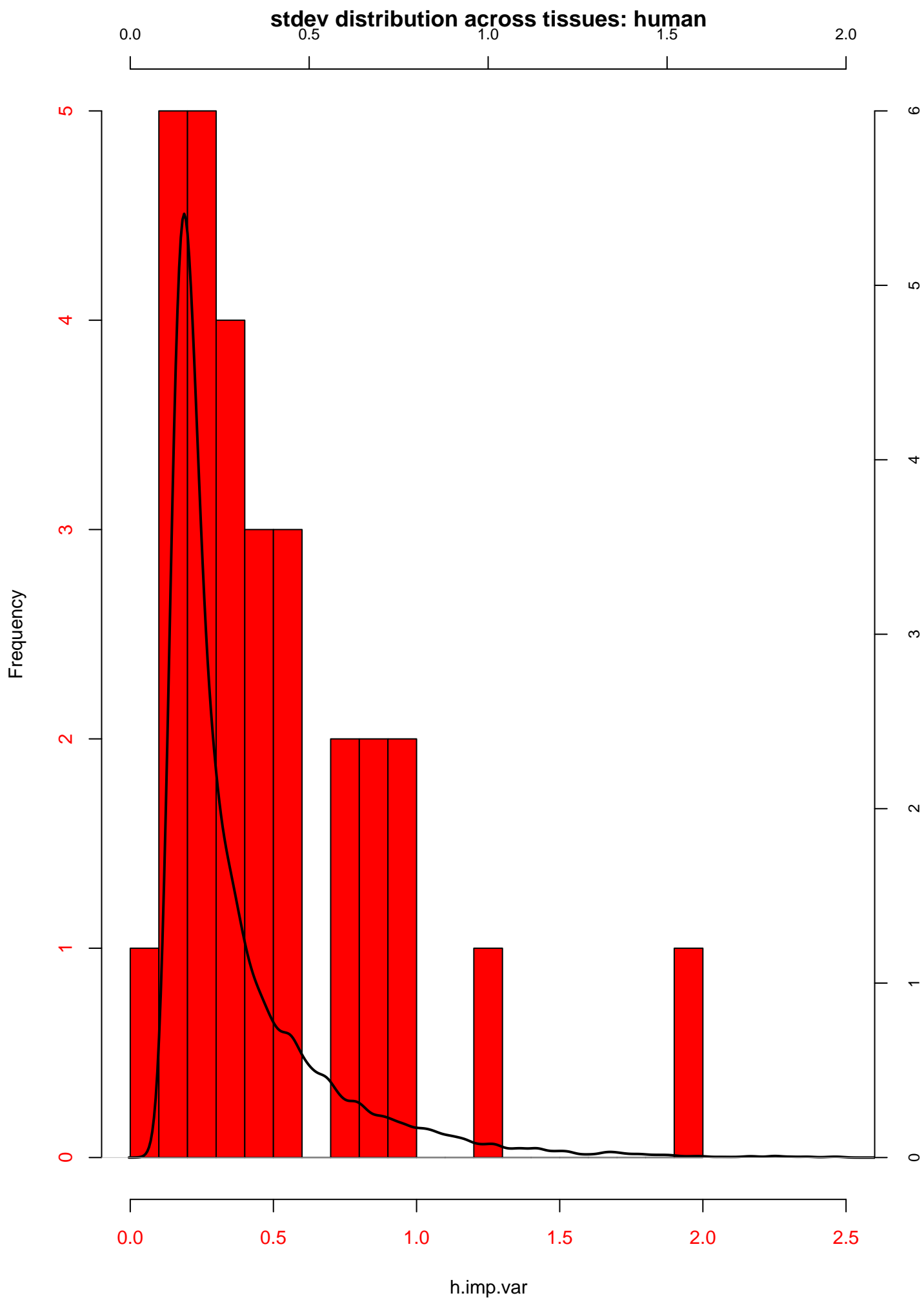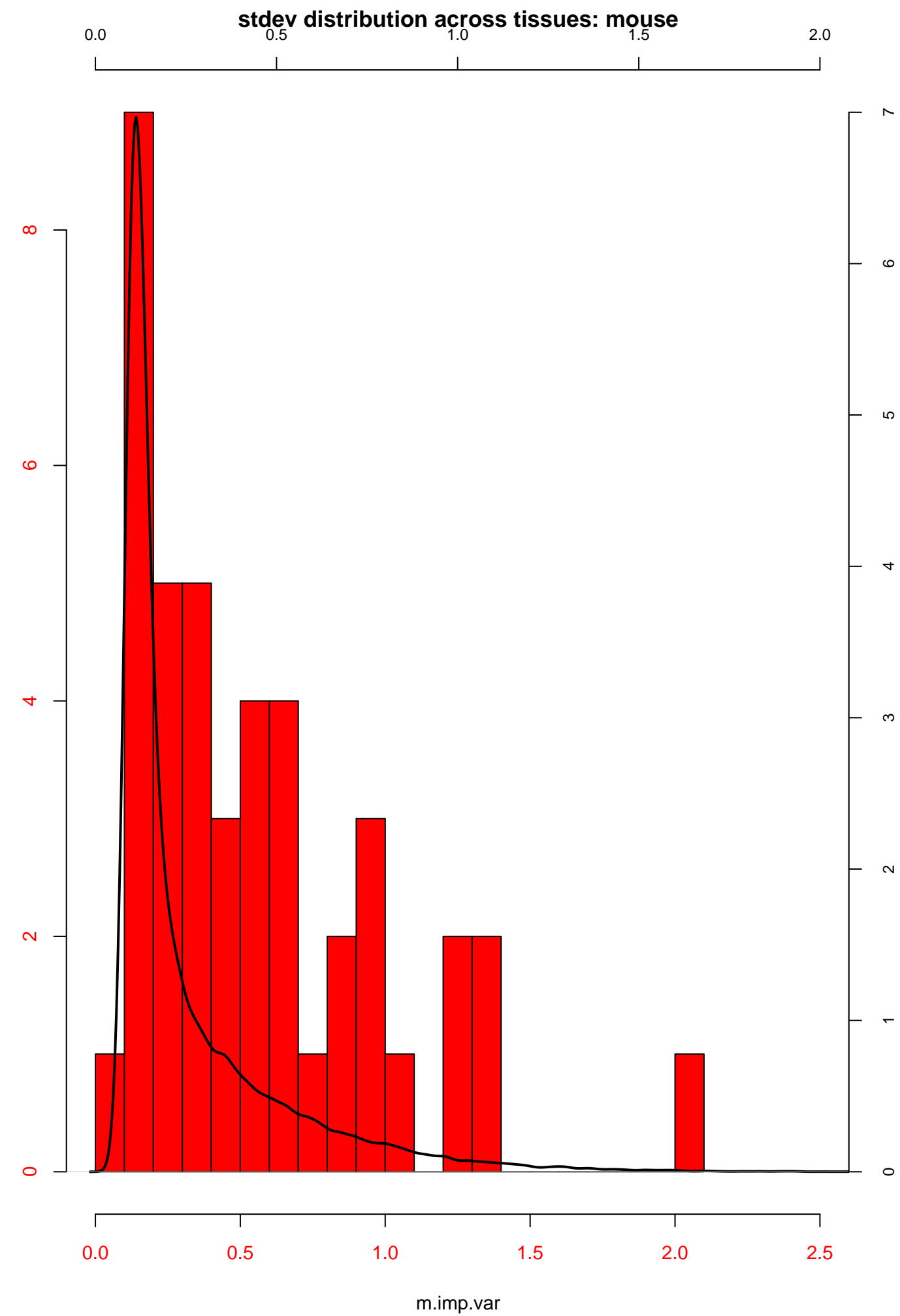

Supplement: Additional file 2 — Distribution of standard deviation across tissues. The left figure displays the distribution for human data whereas the right one shows mouse data. The background distribution, consisting of all genes present on the array but imprinted genes of standard deviation for each gene across tissues is marked in black. The standard deviation of imprinted genes is displayed as red histograms. [file 1471-2164-10-144-S1.pdf]
